# Impact of water, sanitation, and hygiene indicators on enteric viral pathogens among under-5 children in low resource settings

**DOI:** 10.1016/j.scitotenv.2025.178401

**Published:** 2025-01-08

**Authors:** Rina Das, Md. Nasif Hossain, Myron M. Levine, Karen L. Kotloff, Dilruba Nasrin, M. Jahangir Hossain, Richard Omore, Dipika Sur, Tahmeed Ahmed, Robert F. Breiman, A.S.G. Faruque, Matthew C. Freeman

**Affiliations:** a Gangarosa Department of Environmental Health, Rollins School of Public Health, Emory University, Atlanta, GA 30322, USA; b Nutrition Research Division, icddr,b, Dhaka 1212, Bangladesh; c Division of Infectious Diseases and International Health, University of Virginia School of Medicine, Charlottesville, VA, USA; d University of Maryland School of Medicine, Baltimore, MD, USA; e Medical Research Council Unit The Gambia at the London School of Hygiene & Tropical Medicine, Fajara, the Gambia; f Kenya Medical Research Institute (KEMRI), Nairobi, Kenya; g National Institute of Cholera and Enteric Diseases, Kolkata, West Bengal, India; h James P. Grant School of Public Health, BRAC University, Dhaka 1212, Bangladesh; i Department of Global Health, University of Washington, Seattle, WA 98104, USA; j Hubert Department of Global Health, Rollins School of Public Health, Emory University, Atlanta, GA 30322, USA

**Keywords:** Enteric viral pathogens, WASH, GEMS, Under 5 children

## Abstract

Poor water, sanitation, and hygiene (WASH) are the primary risks of exposure to enteric viral infection. Our study aimed to describe the role of WASH conditions and practices as risk factors for enteric viral infections in children under 5. Literature on the risk factors associated with all-cause diarrhea masks the taxa-specific drivers of diarrhea from specific pathogens, limiting the application of relevant control strategies. We analyzed data from children enrolled in the Global Enteric Multicenter Study (GEMS) across seven study sites between December 2007 and March 2011 as cases (moderate-to-severe diarrhea: MSD) and asymptomatic controls. MSD was defined as new and acute diarrhea, with at least one of the following criteria for MSD: dehydration based on the study clinician’s assessment, dysentery, or hospitalization with diarrhea or dysentery. Multiple logistic regression was used to examine the role of water quality, sanitation access, and hygiene facilities on the enteric viral pathogens adjusted for potential covariates. Among MSD symptomatic children (cases), longer water retrieval time (≥15 vs <15 min) was associated with increased Norovirus (aOR 1.33, 95 % CI 1.08–1.64) and Astrovirus (aOR 1.43, 95 % CI 1.01–2.02); scooping as drinking water retrieval method was associated with lower Rotavirus (aOR 0.77, 95 % CI 0.62–0.96), but higher Adenovirus (aOR 2.3, 95 % CI 1.32–4.11) infection compared to non-users. Among asymptomatic children (controls), consumption of non-tube well drinking water was associated with higher Norovirus infection (aOR 1.38, 95 % CI 1.01–1.89). Longer drinking water retrieval time (≥15 vs <15 min) increased Norovirus (aOR 1.47, 95 % CI 1.21–1.78) and Rotavirus (aOR 1.51, 95 % CI 1.20–1.89) infections. Pouring (aOR 0.51, 95 % CI 0.32–0.83) or scooping drinking water with a cup (aOR: 0.52; 95 % CI: 0.32, 0.86) lower Astrovirus infection; restricted water access (aOR 1.57, 95 % CI 1.21–2.02) higher Rotavirus infection. Handwashing before cooking was associated with lower Astrovirus (aOR 0.64, 95 % CI 0.47–0.88) infection in asymptomatic children. Our analysis did not find a significant effect of poor sanitation on different enteric viral pathogens examined. Norovirus and Astrovirus were detected more commonly in sub-Saharan Africa while Rotavirus was less prevalent than South Asia. Though we found statistically significant associations, we did not observe any overall pattern between WASH and enteric viral pathogens. Our findings provide insights to guide further research on targeted interventions for enteric viral pathogens, responsible for a major burden of pediatric diarrhea globally.

## Graphical Abstract

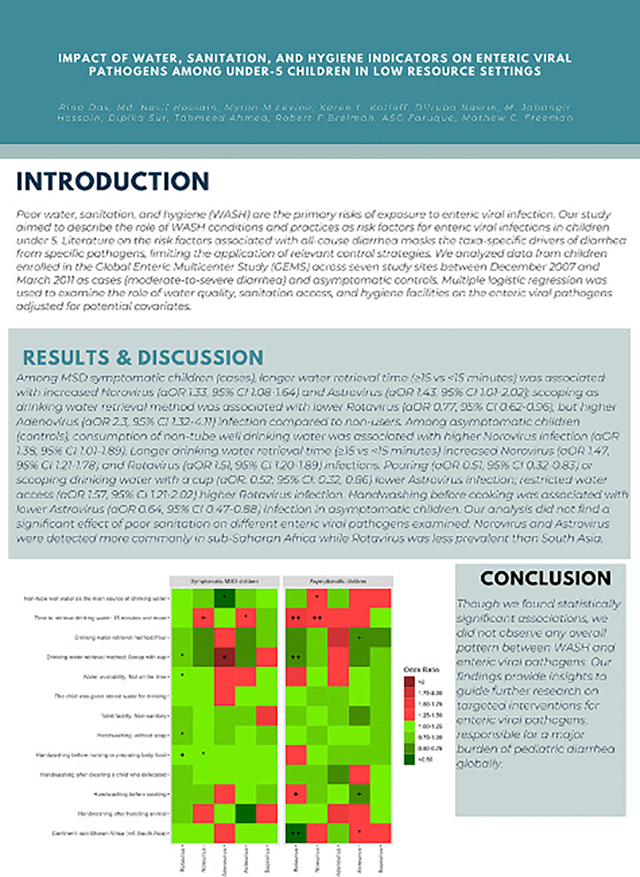

## 1. Introduction

Infections with enteric viral pathogens have a high mortality and morbidity burden, as well as significant social and economic costs (Kolling et al., 2012; Colombara et al., 2016). Although great strides have been made in reducing diarrhea mortality, especially because of the increased use of oral rehydration therapy, diarrhea remains the second leading cause of death in children under 5 years of age, after pneumonia (Walker et al., 2013). It is responsible for an estimated 1.7 billion cases of diarrhea or on average 2.9 episodes/child/year, and an estimated 1.87 million deaths among children under 5 years of age (Boschi-Pinto et al., 2008). Early and repeated infection with enteropathogens is also a risk factor for tropical enteropathy, a condition of an inflammatory condition of the gut that leads to growth shortfalls (Mahmoud et al., 2021; Capone et al., 2023).

According to the WHO/UNICEF Joint Monitoring Program (JMP) (2021), a significant proportion of diarrheal disease burden among children under five years old, estimated at around 88 %, is attributable to inadequate access to safe water, sanitation, and hygiene (WASH) practices, highlighting the critical role of improved WASH interventions in preventing childhood diarrhea (Hailu et al., 2021). The etiology of diarrheal diseases in children under five is multifactorial, often rooted in socioeconomic disparities. Inadequate WASH infrastructure and practices, unsafe human waste disposal, limited access to healthcare and education, coupled with suboptimal dietary intake and poor housing conditions, collectively contribute to the elevated risk of diarrhea in this vulnerable population. Furthermore, the WHO highlights continued disparities in access to basic WASH services, further emphasizing the need for sustained interventions.

According to the Global Enteric Multicenter Study (GEMS), most households across seven sites in South Asia and sub-Saharan Africa (over 93 %) had access to a sanitation facility. However, in Kenya, Mali, Mozambique, and Pakistan, sharing sanitation facilities with other households was identified as a risk factor for diarrhea (Baker et al., 2014); in Bangladesh, most households have an improved water source and sanitation facility (Baker et al., 2014). In Mozambique and India, the presence of soap or ash was more commonly observed in children with asymptomatic infections, and it demonstrated significant protective effects against moderate-to-severe diarrhea (MSD) (Baker et al., 2016). Improvements in water supply and sanitation, if implemented sustainably, will have an important impact on a wide variety of different infectious diseases, and could improve the quality of life of millions of children worldwide, and provide them with a proper start in life.

WASH represents a critical public health intervention domain. Access to clean water, adequate sanitation infrastructure (including safe excreta disposal and wastewater management), and the promotion of hygienic practices, handwashing with soap, constitute the core components of WASH. These interconnected elements are fundamental in mitigating the transmission of enteric pathogens and other infectious diseases, thereby reducing the global burden of diarrheal disease, particularly among vulnerable populations in LMICs (Low- and- middle income contries). At least 1.7 billion people use a drinking water source contaminated with feces; and over 1.5 billion people still do not have basic sanitation services, such as private toilets or latrines (WHO, 2022). In sub-Saharan Africa, 83 % of the individuals living in urban areas in access to improved water sources, while 49 % of the population in rural areas have access. Additionally, urban areas have 43 % of access to improved sanitation facilities, compared to 23 % in rural areas (Baker et al., 2013). Limited and unsafe water, sanitation, and hygiene (WASH) conditions and behaviors are the primary risk factors for enteric pathogen infection (Wolf et al., 2023).

While acute diarrhea is often caused by viral pathogens, limited research has evaluated how WASH impacts specific enteric viral pathogens. Prior studies primarily assessed short-term clinical outcomes among symptomatic children, without characterizing the effect of WASH on viral carriage. The protection conferred by improved WASH against enteric viruses remains unclear. Due to fecal-oral transmission, we investigated the association between WASH conditions and the prevalence of enteric viruses in symptomatic and asymptomatic young children. By examining multiple viruses in both groups and across study settings, our study comprehensively assesses how WASH may reduce enteric viral infections among under under-5 children in low-resource settings. Diarrhea can be caused by viruses, but also other enteric pathogens (e.g., bacteria, protozoa), and non-enteric illnesses (e.g., malaria). Better characterization of taxa-specific pathways of infection can help guide targeted public health interventions and policies to improve WASH and reduce the burden of child morbidity. While viruses are a significant contributor to diarrheal disease, bacterial and protozoan infections account for a substantial proportion of diarrheal-related mortality, particularly in children under five. Rotavirus, remains a leading cause of severe childhood diarrhea globally, although the introduction of rotavirus vaccines has substantially reduced its impact (Organization WH, 2013). Despite this progress, other viral pathogens like norovirus and adenovirus continue to contribute to the burden of diarrheal disease (Lanata et al., 2013). Bacterial pathogens, such as *Shigella* spp. (Livio et al., 2014), enterotoxigenic *Escherichia coli* (ETEC) (Das et al., 2022), and *Vibrio cholerae* (Ali et al., 2015), are responsible for a significant number of diarrheal deaths, particularly in resource-limited settings (Kotloff et al., 2013). Protozoan parasites (Das et al., 2023a), including Cryptosporidium and *Entamoeba histolytica*, also contribute to diarrheal morbidity and mortality.

Our findings add to the limited evidence base on viral etiology in pediatric diarrheal illness and the role of WASH in the transmission of enteric viruses. Unlike prior studies (Rogawski McQuade et al., 2020; Grembi et al., 2023; Colston et al., 2020) that assessed broad WASH interventions or specific outcomes like vaccine immunogenicity or all-cause diarrhea, our study uniquely examines the individual roles of distinct WASH components (water source, sanitation, handwashing practices) as risk factors for specific enteric viral pathogens (rotavirus, norovirus, adenovirus, astrovirus, and sapovirus) in children under five within the GEMS multi-site case-control framework.

Our study aimed to describe the role of WASH situation and practice in sub-Saharan Africa and South Asia as risk factors for enteric viral pathogens in children under 5 years of age. We used data from the Global Enteric Multicenter Study (GEMS), a matched case-control study of MSD in children <5 years old in four sites in sub-Saharan Africa and three sites in South Asia (Kotloff et al., 2012), collected data from symptomatic diarrheal and asymptomatic healthy children on child anthropometry, wealth index, household density, and WASH facilities and practices. We investigated the role of WASH on enteric viral pathogens (rotavirus, norovirus, adenovirus, astrovirus, and sapovirus) among under-5 children in South Asia and sub-Saharan Africa in GEMS.

## 2. Methods

### 2.1. Ethical considerations

Before implementing the GEMS, the local site-specific ethics committees and the University of Maryland School of Medicine ethics committee approved the protocol, clinical procedure, consent forms, CRFs (case report forms), field procedures, and other study-related supporting materials. The signed informed consent forms for the children’s participation in the study were collected from their parents/guardians (both sick cases and healthy controls).

### 2.2. Data collection

GEMS data was collected from ClinEpiDB (Clinical Epidemiology Database), which is a free, online resource that allows us to explore and visualize data from clinical and epidemiological GEMS study. Also, ClinEpiDB has the authority of the GEMS data repository. Data was available upon study team review and approval of our data access request.

### 2.3. Study design

We conducted an analysis of the association between WASH conditions and practices on viral pathogen infection using data from GEMS, a prospective, age-stratified, matched case-control study conducted from December 2007 to February 2011 in seven study sites across Sub-Saharan Africa (The Gambia, Mali, Mozambique, and Kenya) and South Asia (Bangladesh, India, and Pakistan) (Kotloff et al., 2012). Under-5 children of the Demographic Surveillance System (DSS) catchment area, children presenting to the Sentinel Health Center with MSD admitted within 7 days of acute diarrheal onset were considered cases. Age, sex, and community-matched healthy children without diarrhea for the previous 7 days, randomly selected from the same DSS community were enrolled as controls. Controls were enrolled within 2 weeks of case enrolment. Nutritional assessments based on weight, length/height, and mid-upper arm circumference (MUAC) were performed at the time of enrollment (Kotloff et al., 2012). Approximately 60 days after enrollment, GEMS field workers visited the household of each enrolled child (acceptable range, 50–90 days) (Kotloff et al., 2012). Our analysis used the anthropometry data and details of in-between comorbidity data from those follow-up household visits the child was suffering from in the last 60 days after enrollment and if were hospitalized due to (diarrhea, malaria, typhoid, pneumonia, and dysentery) which were documented during hospital discharge.

**Fig. 1.**
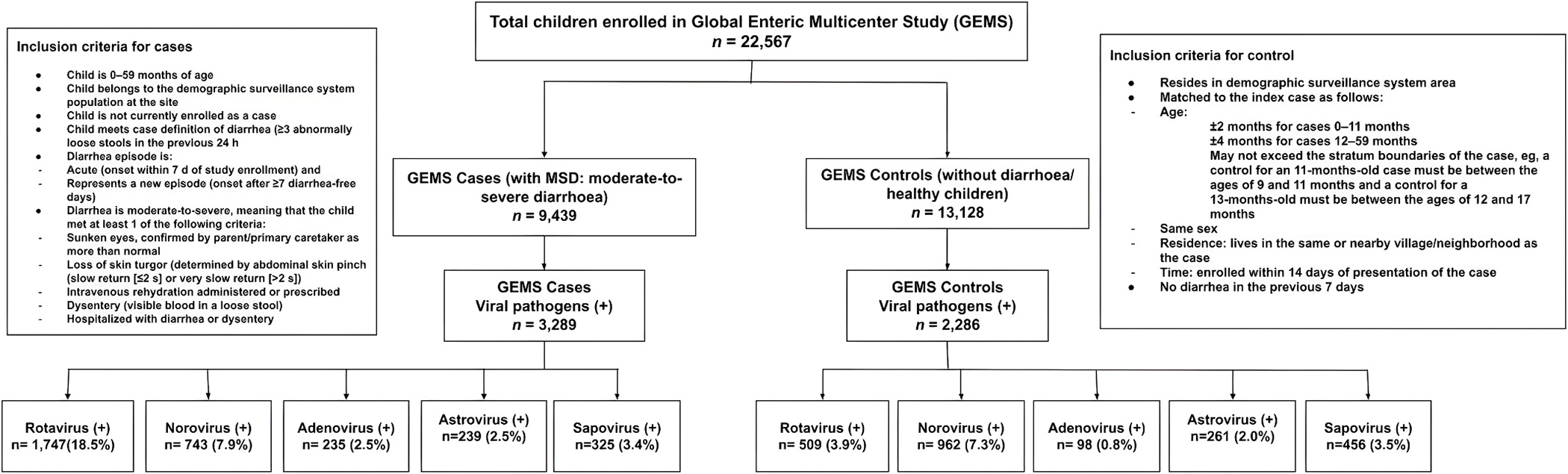
Study flow diagram.

For this analysis, we included 22,567 children. Among all enrolled children, 3289 were symptomatic MSD children and 2286 were asymptomatic healthy children who were positive for enteric viral pathogens (Fig. 1).

### 2.4. Outcome variables

*Outcome variables* were the presence of enteric viral pathogens (Rotavirus, Norovirus, Adenovirus, Astrovirus, Sapovirus) in GEMS cases (symptomatic diarrhea children) and controls (asymptomatic children without diarrhea).

### 2.5. Exposure variables

#### 2.5.1. WASH conditions and practices

Data on WASH was collected through initial assessments and follow-up visits, including information on water sources, fecal waste disposal methods, and hygiene practices such as handwashing. Variables were self-reported and also based on direct observations: hand washing practice by the primary caregivers (before nursing or preparing baby food; after cleaning a child who defecated; before cooking and after handling animal), the main source of drinking water (tube well water/non-tube well water), the child was given stored drinking water (yes/no), time to retrieve drinking water (<15 min vs 15 min or more), fetch drinking water everyday (yes/no), number of water trips per day, water availability (all the time vs not all the time: several hours every day to less frequently than a few times per week), drinking water retrieval method (pour/scoop with cup/scoop with a ladle), water storage container covered or not, having narrow/wide open mouth, sanitation facilities (toilet facility for disposal of human fecal waste: sanitary/semi sanitary vs. non-sanitary), and the use of handwashing materials (water with soap/without soap).

#### 2.5.2. Moderate-to-severe diarrhea (MSD)

MSD was defined as new and acute diarrhea (≥3 abnormally loose stools within the past 24 h) that started within the previous 7 days following at least 7 diarrhea-free days, with at least one of the following criteria for MSD: dehydration based on the study clinician’s assessment (sunken eyes; decreased skin turgor; or intravenous rehydration administered or prescribed); dysentery (visible blood in stools reported by the mother or observed by the study team); or hospitalization with diarrhea or dysentery.

### 2.6. Stratification variables

Given that GEMS was a case-control study, we present stratified models based on Moderate-to-severe Diarrhea (MSD) status. MSD (case) was defined as new and acute diarrhea (≥3 abnormally loose stools within the past 24 h that started within the previous 7 days following at least 7 diarrhea-free days), with at least one of the following criteria for MSD: dehydration based on the study clinician’s assessment (sunken eyes; decreased skin turgor; or intravenous rehydration administered or prescribed); dysentery (visible blood in stools reported by the mother or observed by the study team); or hospitalization with diarrhea or dysentery (Kotloff et al., 2012) (Fig. 1). As part of the study design, MSD children were matched with asymptomatic children (controls) by age, sex, and community-matched healthy children without diarrhea for the previous 7 days randomly selected from the same DSS community were enrolled as asymptomatic children (Kotloff et al., 2013) (Fig. 1).

### 2.7. Covariates

#### 2.7.1. Breastfeeding

Breastfed refers to both exclusive and partially breastfed children under 2.

#### 2.7.2. Sociodemographic information

Using principal component analysis, households were categorized based on the wealth quintiles as socioeconomic status (SES) (poor, lower-middle, middle, upper-middle, and rich) (Filmer and Pritchett, 2001). The survey includes data on asset indicators that can be grouped into three types: household ownership of consumer durables (clock/watch, bicycle, radio, television, bicycle, sewing machine, refrigerator, car); characteristics of the household’s dwelling (about toilet facilities, the source of drinking water, rooms in the dwelling, building materials used, and the main source of lighting and cooking); and household land ownership (Filmer and Pritchett, 2001). Children were sorted by the asset index and established cutoff values for percentiles of the population. Then the households were assigned to a group based on their value on the index. For expository convenience, GEMS refers to the bottom 20 % as “poor” the next 20 % as “lower-middle”, the next 20 % as “middle”, the next 20 % as “upper-middle” and the top 20 % as “rich” but this classification does not follow any of the usual definitions of poverty (Filmer and Pritchett, 2001).

### 2.8. Statistical analysis

We reported the child and household-level characteristics by using mean and standard deviation for continuous variables and frequency as a percentage for categorical variables to summarize the data. To assess the association between the enteric viral pathogens at baseline and WASH situations and practice, we used a multiple logistic regression model, where the explanatory variables were WASH status and practice; and the outcome variables were the presence of enteric viral pathogens. Based on the bivariate analysis and literature review we include all the factors and age, gender, and continent as covariates in the final multiple logistic regression model. The variance inflation factor (VIF) was calculated to detect multicollinearity, and no variable with a VIF value >5 was identified in the final model. We estimated the β coefficient and its 95 % CI to describe the precision of the point estimate. Variables related to WASH indicators with >10 % missing values were omitted from the multiple logistic regression model. The P-value of <0.05 was considered statistically significant and all analyses were conducted in R v.4.3.1.

## 3. Results

The percentage of symptomatic MSD children aged 24–59 months (23.4 %) was significantly lower than that of asymptomatic children (29.5 %) in the same age group (p = 0.01). Baseline anthropometric measurements (WAZ: weight for age z score mean ± SD: − 1.08 ± 1.32 vs − 1.51 ± 1.39 and WHZ: weight for height z score − 0.47 ± 1.49 vs − 1.06 ± 1.49) showed a statistical difference (p-value <0.001) between asymptomatic and symptomatic children respectively (Table 1) which was also reported previously (Kotloff et al., 2013; Das et al., 2023b). Symptomatic MSD children were more often (82.3 %) using non-tube well water as the main source of drinking water compared to asymptomatic healthy children (77.2 %, p-value <0.001). Non-sanitary latrines were more common among the households of asymptomatic children compared to symptomatic MSD children (6.4 % vs 4.9 %; p < 0.001). *Cryptosporidium* (11.9 % vs 6.4 %), *E. histolytica* (2.9 % vs 2.3 %), ETEC: enterotoxigenic *E. coli* (11.3 % vs 7.4 %), Rotavirus (18.5 % vs 3.9 %), Adenovirus (2.5 % vs 0.7 %) and *Shigella* (11.8 % vs 1.8 %) were more frequently detected among the children exhibiting symptoms in comparison to asymptomatic children (p-value <0.001 for each pathogen) respectively and described previously (Das et al., 2023b; Nasrin et al., 2021) (Table 2).

**Fig. 2.**
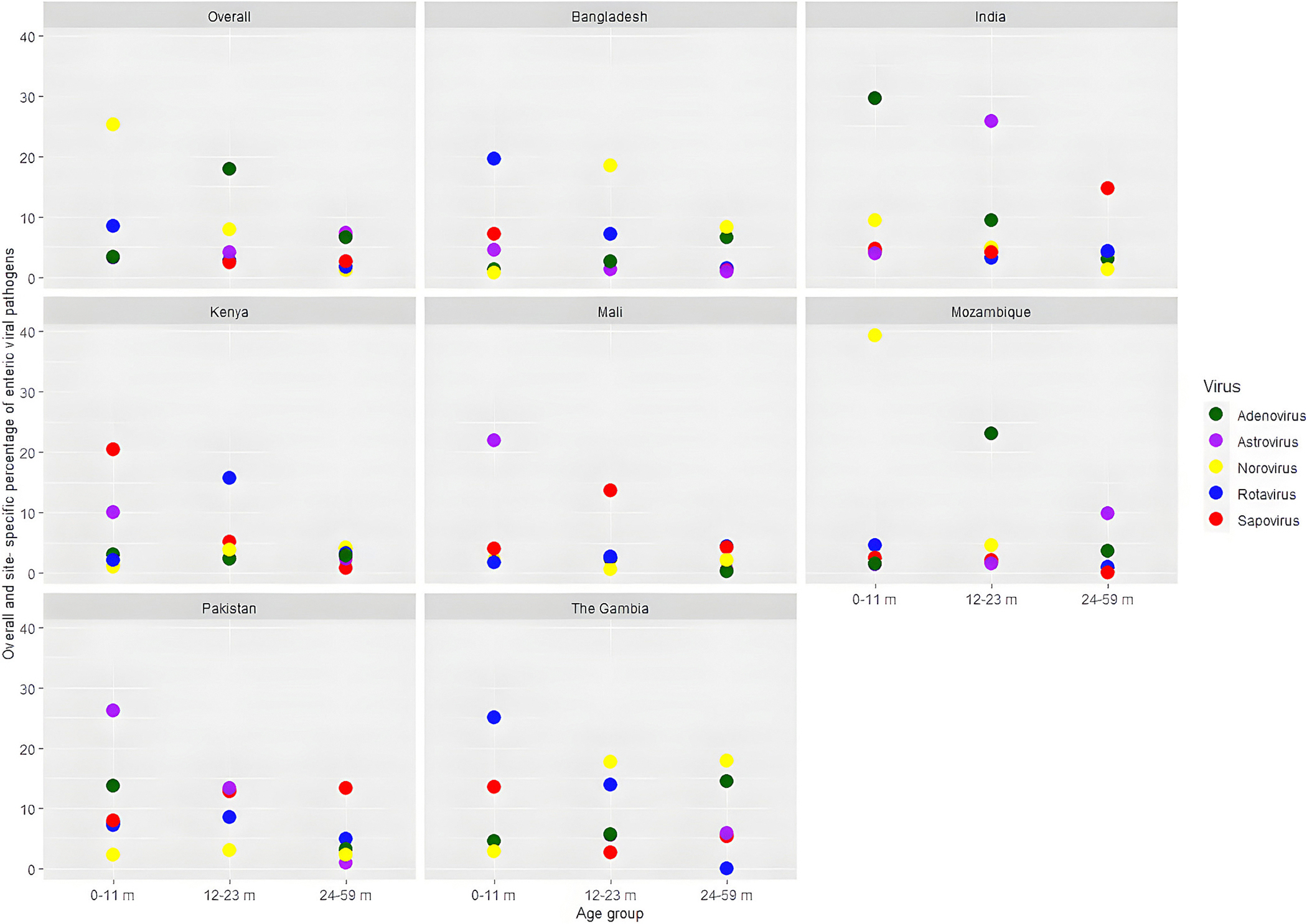
Overall and site-specific percentage of enteric viral pathogens isolated from the stools of symptomatic MSD children.

**Fig. 3.**
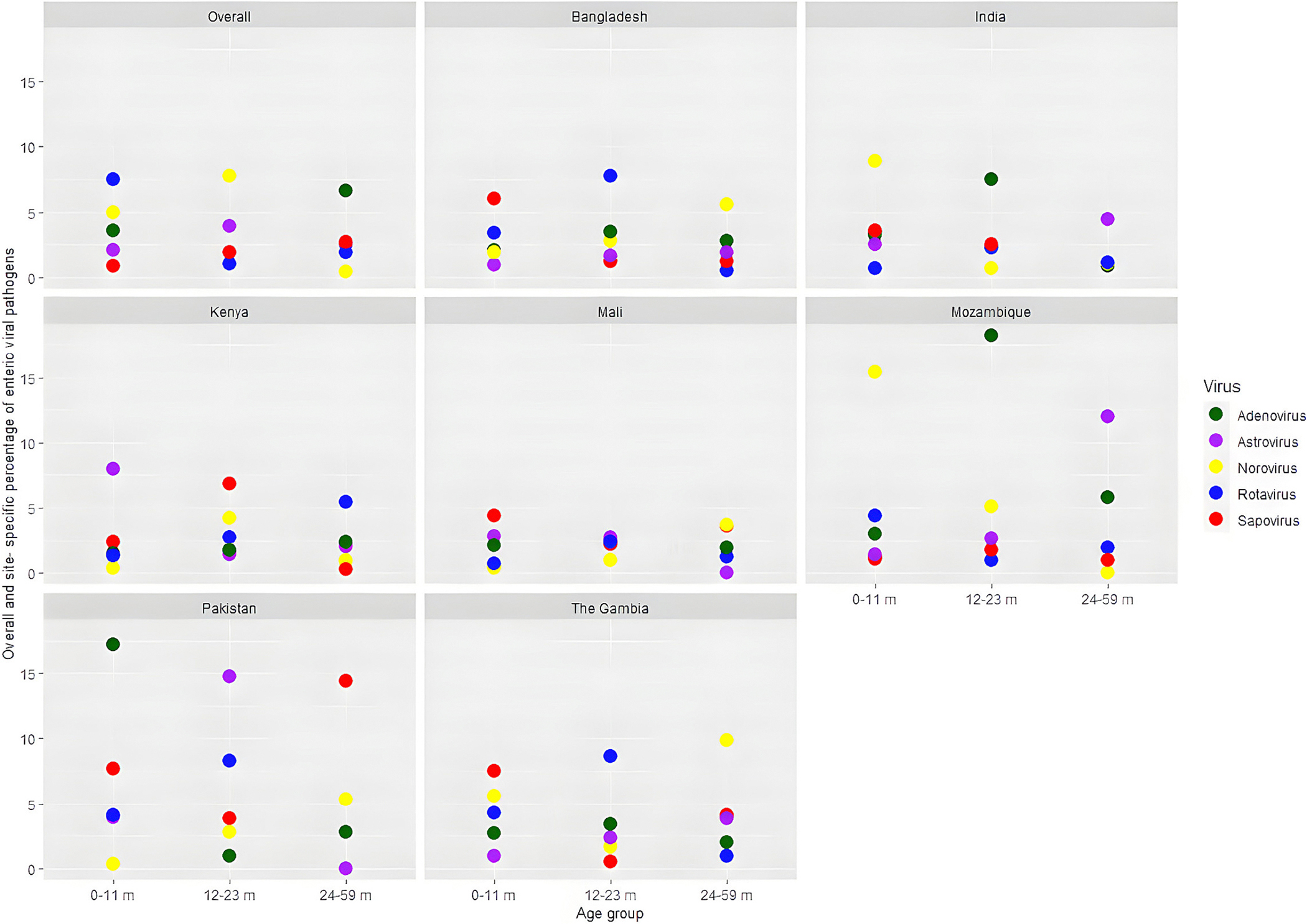
Overall and site-specific percentage of enteric viral pathogens isolated from the stools of asymptomatic healthy children.

All five enteric viral pathogens were more often (40–50 %) detected among younger children (age 0–11 months). Among the symptomatic children, rotavirus was detected in 1747 (18.5 %), norovirus in 743 (7.9 %), adenovirus in 235 (2.5 %), astrovirus in 239 (2.5 %), and sapovirus in 325 (3.4 %), also described elsewhere (Kotloff et al., 2013). Overall and site-specific percentages of enteric viral pathogens isolated from the stools of symptomatic MSD and asymptomatic healthy children are shown in Figs. 2 and 3.

The baseline demographic characteristics of the symptomatic MSD children having stool positive for different viral pathogens in South Asia and sub-Saharan Africa are presented in S1 and S2 Tables.

Among the asymptomatic children, two-thirds of the children received treated water for drinking; >90 % of the households had improved toilet facilities, and almost 70 % of caregivers used water and soap for handwashing compared to the children who were negative for enteric viral pathogens. Children positive for all five enteric viral pathogens (Rotavirus, Norovirus, Astrovirus, Adenovirus, Sapovirus), >42 % needed <15 min to retrieve drinking water; >60 % of children had all-time water availability at households compared to the children negative for viral pathogens (Table 3).

**Fig. 4.**
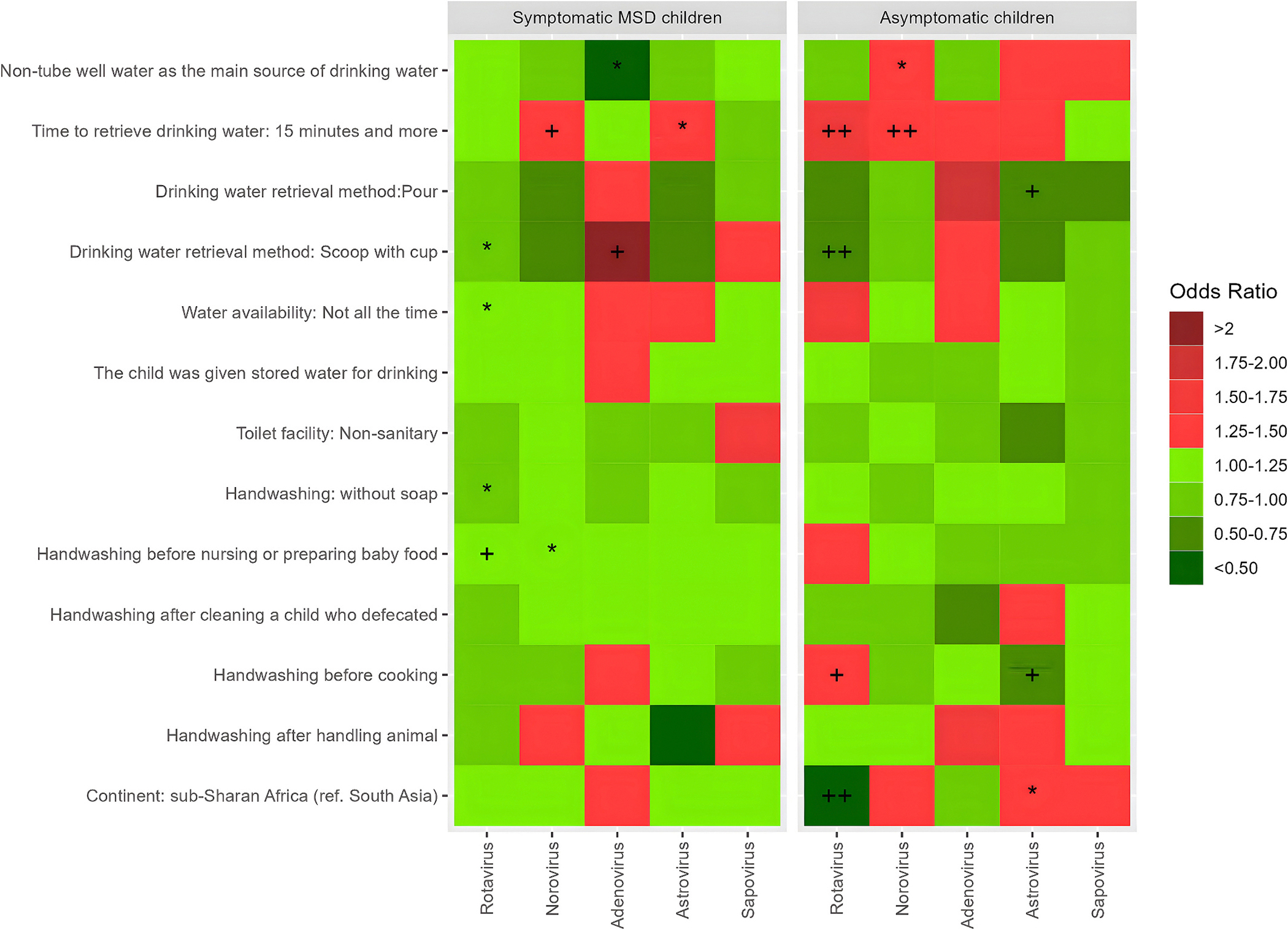
Heat map of adjusted odds ratios of associations between water, sanitation, and hygiene (WASH) covariates and enteric viral pathogens among symptomatic MSD and asymptomatic under 5 children. [* indicates statistical significance at p < 0.05, + indicates statistical significance at p < 0.01, ++ indicates statistical significance at p < 0.001.]

Among the asymptomatic children, Norovirus (aOR 0.46, 95 % CI 0.35–0.60) and Astrovirus (aOR 1.46, 95 % CI 1.17–1.82) were detected more commonly in sub-Saharan Africa while Rotavirus (aOR 1.47, 95 % CI 1.01–2.17) was less prevalent compared to South Asia (Fig. 4). No significant geographical variation was observed among the MSD children in the present analysis (Fig. 4).

### 3.1. Associations between WASH and enteric viral pathogens

#### 3.1.1. Rotavirus

Among symptomatic MSD children, using the scoop as the method of retrieving drinking water decreased the odds of Rotavirus infection (aOR 0.77, 95 % CI 0.62–0.96) compared to non-scoop users; water unavailability (not all the time) was associated with higher Rotaviral infection (aOR 1.2, 95 % CI 1.01–1.42) compared to who had all the time water availability at household. Primary caretakers’ handwashing practice without soap (aOR 0.85, 95 % CI 0.73–0.99) was associated with reduced odds of a child’s Rotavirus infection compared to soap user. However, caregiver handwashing before infant feeding increased the odds of Rotavirus infection (aOR 1.21, 95 % CI 1.06–1.4) compared to the caregiver who did not wash hands (Fig. 4). Among the asymptomatic children, an increased water retrieval time (of 15 min or more) causes increased odds of Rotavirus infection (aOR 1.51, 95 % CI 1.20–1.89) compared to the retrieval time (<15 min). Restricted water access (water availability at household: not all the time) increased the odds of Rotavirus infection (aOR 1.57, 95 % CI 1.21–2.02) compared to those who had non-restricted water access. Handwashing before cooking and preparing baby food increased the odds of Rotavirus infection (aOR 1.48, 95 % CI 1.18–1.85) and (aOR 1.50, 95 % CI 1.17–1.94) respectively. Scooping drinking water with a cup (aOR 0.51, 95 % CI 0.35–0.73) reduced the odds compared to non-scoop users (Fig. 4).

#### 3.1.2. Norovirus

Among the symptomatic MSD children, longer water retrieval time (of 15 min or more) was associated with higher odds of Norovirus infection (aOR 1.33, 95 % CI 1.08–1.64) compared to a shorter retrieval time (of <15 min). Using a scoop as the method of retrieving drinking water decreased the odds of Norovirus infection (aOR 0.75, 95 % CI 0.55–1.04) compared to non-scoop users. However, caregivers’ handwashing before infant feeding increased the odds of Norovirus infection (aOR 1.24, 95 % CI 1.01–1.52) (Table 3) compared to the caregivers who did not wash hands prior to infant feeding. In Fig. 4, asymptomatic children consuming non-tube well drinking water was associated with higher odds of Norovirus infection (aOR 1.38, 95 % CI 1.01–1.89) compared to tube well water consumer. A retrieval time of 15 min or more increased the odds of Norovirus infection (aOR 1.47, 95 % CI 1.21–1.78) compared to a retrieval time of <15 min among asymptomatic children.

#### 3.1.3. Adenovirus

Among the symptomatic MSD children, using a scoop as the method of retrieving drinking water increased the odds of Adenovirus infection (aOR 2.3, 95 % CI 1.32–4.11) compared to the non-scoop users. On the other hand, consuming drinking water from a non-tube well source decreased the odds of Adenovirus infection (aOR 0.47, 95 % CI 0.27–0.86) compared to tube well water source (Fig. 4) among the symptomatic MSD children.

#### 3.1.4. Astrovirus

Symptomatic MSD children, who had longer water retrieval time (of 15 min or more) were associated with higher odds of Astrovirus infection (aOR 1.43, 95 % CI 1.01–2.02), compared to a retrieval time of <15 min (Table 3). Among the asymptomatic children, pouring (aOR 0.51, 95 % CI 0.32–0.83) or scooping drinking water with a cup (aOR 0.52, 95 % CI 0.32–0.86) reduced the odds of Astrovirus infection. Handwashing before cooking reduced the odds of Astrovirus infection (aOR 0.64, 95 % CI 0.47–0.88) compared to those who did not wash hands before cooking (Fig. 4 among asymptomatic children).

Sanitation access was not statistically associated with any virus (Fig. 4). Children who used non-sanitary latrine had higher odds of symptomatic Norovirus infection (aOR: 1.07, 95 % CI 0.69, 1.6) and symptomatic Sapovirus infection (aOR 1.62, 95 % CI 0.88–2.76) compared to sanitary/semi sanitary toilet users. Among the asymptomatic children who used non sanitary toilet facilities had increased odds of Norovirus (aOR 1.07, 95%CI 0.76, 1.46) infection compared to improved toilet users.

## 4. Discussion

We quantified the association between WASH conditions and laboratory-confirmed viral infections among children with and without MSD in seven sites in sub-Saharan Africa and Asia. We did not observe any consistent patterns of associations between specific WASH exposures and enteric viral pathogens among symptomatic MSD and asymptomatic children under 5 years of age across the different study sites in our analysis. For the symptomatic MSD children, longer water retrieval times (≥15 min) were associated with higher infection with Rotavirus, Norovirus, and Astrovirus. The use of proper scooping techniques for water retrieval was associated with lower infection with Rotavirus, while non-scoop use increased the risk. Handwashing before infant feeding was associated with higher Rotavirus and Norovirus infection. For asymptomatic children, longer water retrieval times (≥15 min) were associated with high infection with Rotavirus and Norovirus. Inadequate/restricted water access was also associated with higher Rotavirus infection. Drinking non-tube well water was associated with higher Norovirus infection. Safe water handling methods like proper pouring or scooping and handwashing before food preparation were associated with lower Astrovirus infection. Other associations between WASH conditions and viral infections were not statistically significant. The lack of clear trends in the association between WASH conditions and viral infection for symptomatic or asymptomatic MSD cases is probably due to multiple interrelated WASH and socioeconomic factors impacting transmission risk simultaneously, it is hard to isolate the independent effect of a single WASH variable. And this underscores a weak evidence base for the impact of specific WASH behaviors and practices or access to specific WASH facilities on viral infections. It also highlights the challenges in exposure measurement within the WASH sector (Goddard et al., 2020) and challenges in elucidating temporality between exposures and outcomes for enteric diseases. Below we discuss pathogen-specific considerations relevant to these findings.

Supplying water from non-tube well sources is a potential source of exposure for enteric viruses that can cause enteric illness. Our analysis suggests that non-tube well water is a likely route of exposure and transmission for Norovirus. The asymptomatic nature of most Norovirus infections also allows for potential transmission from infected individuals via water sources. In contrast, a protective effect was seen for symptomatic Adenovirus infection in those who used non-tube well water. We hypothesize several virological and epidemiological factors may explain the divergent relationships observed. Norovirus exhibits enhanced aquatic persistence relative to Adenovirus, potentially facilitating increased transmission through water ingestion (Yang et al., 2022). Meanwhile, interpersonal contact represents the major dissemination route for Adenovirus, diminishing the influence of water quality on infection risk (Morawska, 2005). Physicochemical attributes of Adenovirus likewise promote more rapid particle sedimentation from water, thereby limiting opportunities for oral uptake from this exposure source (Scalize et al., 2021). Our study also revealed that drinking non-tube well water, likely treated before consumption, had a protective effect against enteric viral infections. Despite being considered improved water sources, tube wells in flood-prone rural areas remain vulnerable to fecal contamination (Luby et al., 2008). Ceramic filtration, commonly used for water treatment in certain GMES sites, is highly effective in removing fecal bacterial species but less effective in eliminating viral enteric pathogens from water. According to CDC, viral pathogens can be present in various water sources, including tube wells contaminated by feces from infected individuals. Contamination can occur through sewage overflows, malfunctioning sewage systems, and polluted storm-water runoff, with tube wells being particularly susceptible to such contamination after floods, especially if they are shallow, recently constructed, or submerged by floodwater (Cortese and Parashar, 2009).

We sought to elucidate associations between household water accessibility metrics and the risk of acquiring enteric viral pathogens. Adequate water accessibility facilitates optimal hygienic practices crucial for curbing fecal-oral disease transmission. Our results demonstrated longer water fetch durations and constrained water accessibility correlated with susceptibility to Rotavirus, Norovirus, and Astrovirus infections. While minimal water accessibility was present across communities, quantities, and qualities available may have inadequately met basic needs. Proximities of water sources to domiciles also varied considerably (Anthonj et al., 2024). Insufficient water provisioning conceivably compromised hygienic behaviors like handwashing, a mainstay barrier to enteric viral dissemination. Augmented household water allotments characteristically engender improved hygienic standards. Nevertheless, numerous settings harbor water sources contaminated with microbial or chemical constituents, rendering consumed volumes unpotable. More remote sources exhibiting longer hydraulic retention periods afforded increased fecal matter infiltration (Raj and Jamwal, 2024). Prolonged carry durations moreover permitted opportunities for stored water to become re-contaminated via contact events during collection, transport, and utilization prior to ingestion, potentially propagating enteric viral pathogenicity (Ihekweme et al., 2023). It is plausible that these limitations in water access and quality have underpinned our findings.

Water retrieval methods play an important role in enteric viral transmission among communities that share water sources (Shen et al., 2024). In resource-limited settings of sub-Saharan Africa (Baddianaah et al., 2024) and Asia (Nwadike et al., 2024), long retrieval times from open, unprotected sources enable viral persistence and potential person-to-person transmission (Nwadike et al., 2024). Intriguingly, our findings showed the use of proper scooping techniques during retrieval was associated with lower odds of rotavirus and norovirus infection, contrasting with non-scoop use which increased rotavirus risk. Furthermore, retrieval methods involving direct pouring from sealed containers, and avoiding physical contact between the retrieval utensil and source water, demonstrated similar protective effects against rotavirus and norovirus. This suggests retrieval approaches that minimize direct utensil-water contact may help curb person-to-person viral transmission reliant on shared water infrastructure. These findings contribute to the considerable evidence (Matsinhe et al., 2014; Wright et al., 2004) that improved handwashing and improved access at the point of use could help mitigate the diarrhoeal pathogen burden in resource-constrained communities (Eshcol et al., 2009). Further research is still needed to understand the mechanisms influencing these observational associations.

While handwashing without soap was associated with lower Rotavirus infections, other enteric viruses commonly transmitted via the fecal-oral route, such as norovirus, are more resistant to simple washing and require the use of soap to be effectively removed from hands. For instance, certain Norovirus and Adenovirus strains can remain infectious on hands even after water-only washing. As Rotavirus is less tolerant of water alone compared to these other enteric viruses, handwashing promotion may prove an effective strategy for preventing Rotavirus infections through mechanical removal, despite the lack of soap. Moreover, the observed reduction in rotavirus infections with handwashing, even without soap, likely reflects the removal of substantial viral load through mechanical rinsing, sufficient to interrupt transmission for this relatively labile virus. However, the greater resilience of norovirus necessitates the virucidal properties of soap for effective hand hygiene, highlighting the importance of considering pathogen-specific characteristics when interpreting handwashing efficacy. Further studies, including water quality and microbial contamination levels, may confound these observations and should be incorporated into analyses to accurately assess the independent contribution of soap to the prevention of infection.

Interestingly, handwashing before cooking had a protective effect on Astrovirus infection, while handwashing practices Before nursing/preparing baby food and before cooking had a detrimental effect on Rotaviral infections, likely due to ineffective handwashing without soap. While handwashing practices in some regions of Asia could be improved, studies have shown that access to safe water and sanitation remains limited for vulnerable populations such as female residents of rural and urban slum communities, contributing to inadequate hand hygiene after exposure to fecal contamination (Das et al., 2021). It was also found that caregivers expressed a preference for soap when it came to handwashing; however, due to cost considerations, they often resorted to using ash or a combination of detergent and ash as a more affordable alternative (Baker et al., 2014). Furthermore, water contamination may confound the observed relationship between handwashing and infant Rotavirus and Norovirus infections. In settings with contaminated water sources, handwashing, even with soap, could inadvertently introduce pathogens to infants through direct ingestion or contamination of food/utensils, potentially offsetting the benefits of removing pathogens already on hands. This is supported by studies (Fewtrell et al., 2005; Clasen et al., 2015; Luby et al., 2005) demonstrating the critical role of water quality in diarrheal disease prevention and the enhanced effectiveness of handwashing when combined with improved water supply. Therefore, future research must account for water quality through microbiological assessment, stratified analyses, and integrated interventions to accurately evaluate the impact of handwashing on these infections. These measures will enable a more accurate evaluation of handwashing’s true impact on infant infections and should inform future research, while current public health messaging should continue to emphasize handwashing with the cleanest available water.

In our analysis, the use of non-sanitary latrines did not affect the children with viral pathogens, potentially due to sharing sanitation facilities with other household members. Sharing facilities may pose challenges in practicing caregivers’ hygienic behaviors, such as handwashing after defecation, which could increase the risk of viral infections in children (Heijnen et al., 2015). This lack of an observed effect contrasts with evidence from previous research demonstrating that improved sanitation is linked to lower burdens of bacterial and protozoal enteric pathogens (Muyyarikkandy et al., 2024). We hypothesize the discrepancy may relate to fundamental differences in hygiene behaviors such as handwashing after defecation present challenges in households sharing sanitation infrastructure, potentially facilitating fecal-oral viral spread among cohabiting individuals. In the MAL-ED study, they showed that the presence of a household toilet was associated with a lower risk of bacterial and protozoal enteric infections, but not viral infections (Berendes et al., 2017). Similarly, A previous study conducted in urban India demonstrated that viral pathogens have lower infectious doses and higher levels of shedding compared to bacteria (Berendes et al., 2017). Infants, who are more susceptible to rotavirus, are unlikely to acquire the infection through the toilet or handwashing facilities since they are not yet at an age to use them. Instead, they are more likely to be infected through contact with caregivers, other infected children, or consumption of contaminated food and water (Berendes et al., 2017). Further investigation of viral-specific transmission dynamics is warranted to better explain these observations.

Certain associations observed in our analysis were unexpected and may be due to chance, as they contradict known risk factors like handwashing and the use of drinking water from a non-tube well source. We could not observe any association between water storage on viral infections in our analysis. Safe storage of treated water is necessary to prevent recontamination through unsafe water and previous research in rural Bangladesh showed that safe water storage with narrow-mouthed containers effectively reduced diarrhea cases by up to 30 % in children, although this study focused only on *Escherichia coli*, leaving the impact of safe storage on viral contamination unclear (Ercumen et al., 2015). Traditional preventive measures, like handwashing and safe water storage, may be less effective than newer approaches such as the rotavirus vaccine, making standardization challenging in this study (Mokomane and Kasvosve, 2018). Our study offers valuable initial data that describes these relationships, serving as a foundation for future research to further explore and understand these associations’ clinical, epidemiological, and public health implications in more specific terms.

Notably, In South Asia, Rotaviral infection is more common than in sub-Saharan Africa. The higher temperature and humidity in parts of South Asia may enable the virus to survive longer in the environment, facilitating transmission. Sub-Saharan Africa has more variable climates which may impair viral survival at certain times or places. Another reason was there might be the study sites of South Asian countries (Bangladesh, India, and Pakistan) have higher population densities on average, allowing Rotavirus to spread more readily due to close contact between infected and susceptible individuals. Persistent gut inflammation from fecal contamination is more common in South Asia, potentially increasing susceptibility to rotavirus infection and severity of disease. On the other hand, Norovirus and Astrovirus infections are more common among asymptomatic children in sub-Saharan Africa than in South Asia, and differences in childhood immunology and gut microbiome between the populations might be the cause behind this finding. Factors like malaria, and HIV prevalence, may prime the immune system and gut flora in sub-Saharan Africa which could impact virus shedding and disease severity.

There are several potential reasons why we did not observe any consistent associations between specific WASH exposures and viral infections across the study population. Enteric virus transmission dynamics are likely influenced by significant geographic and cultural variation, thus WASH behavior and practice shown to reduce risk in one setting may not translate elsewhere. Additionally, differences in viral strains with distinct routes and environmental stability further complicate the determination of overall pathogen relationships. Finally, exposure impacts on symptomatic versus asymptomatic shedding/infection may diverge in complex site-specific patterns. Thus, the enteric virus transmission complexity, coupled with limitations of cross-sectional analyses across diverse populations and environments, hindered the identification of uniform WASH associations in our study.

We had a large, randomly sampled population, data on direct observation of the WASH practices during the household visit and conducted high-quality laboratory procedures.

## 5. Conclusion

Our study identified several individual WASH factors associated with enteric viral infection among children with and without diarrhea in low-income settings. Improved water access, storage, and hygiene practices thus have the potential to reduce enteric viral transmission. However, we did not find any overarching trends that could provide useful guidance to programs and policies for the control of viral pathogens. We observed some divergent associations with virus and symptoms status, warranting future research. The relationships observed between individual WASH factors and enteric viral infection underscores the need for more rigorously designed epidemiological studies to provide stronger causal insight into optimally effective multi-pronged community WASH programs. This would allow guidelines to be established, facilitating targeted improvements across the WASH sector aimed at reducing the burden of viral disease among children in resource-limited communities.

## Supplementary Material

Supplementary file 2

Supplementary file 3

Supplementary file 4

Supplementary file 1

## Figures and Tables

**Table 1:** Baseline characteristics of the asymptomatic and symptomatic MSD children in South Asia and sub-Saharan Africa.

Characteristics Total (n = 22,567)	Asymptomatic children n = 13,128	Symptomatic MSD children n = 9439	P value

Age group			
0–11 m	4878 (37.2)	4030 (42.7)	Ref.
12–23 m	4381 (33.4)	3205 (33.9)	0.18
24–59 m	3870 (29.5)	2205 (23.4)	0.01
Gender (girl)	5651 (43.0)	4095 (43.4)	0.39
Baseline anthropometry			
HAZ/LAZ^a^	−1.34 ± 1.31	−1.34 ± 1.37	0.67
WAZ^a^	−1.08 ± 1.32	−1.51 ± 1.39	<0.001
WHZ^a^	−0.47 ± 1.49	−1.06 ± 1.49	<0.001
Breastfeeding status			
Breastfed	9039 (68.9)	6741 (71.4)	Ref.
Not breastfed	4090 (31.2)	2698 (28.6)	0.13
Primary caretaker’s education			
Illiterate	5168 (39.4)	4016 (42.7)	Ref.
Literate	7935 (60.6)	5386 (57.3)	0.17
Wealth index			
Poorest	2510 (19.1)	2027 (21.5)	Ref.
Lower middle	2590 (19.7)	1813 (19.2)	0.13
Middle	2834 (21.6)	1993 (21.1)	0.23
Upper middle	2522 (19.2)	1780 (18.9)	0.28
Richest	2672 (20.4)	1821 (19.3)	0.23
Water, sanitation, and hygiene (WASH) condition and practice			
The main source of drinking water			
Tube well water	2994 (22.8)	1675 (17.7)	Ref.
Non-tube well water	10,135 (77.2)	7765 (82.3)	<0.001
Handwashing material			
With soap and water	9762 (74.4)	7131 (75.6)	Ref.
Without soap	3365 (25.6)	2308 (24.5)	0.64
Handwashing practice			
Before nursing a child	5133 (39.1)	3683 (39.0)	0.99
After cleaning a child who defecated	6189 (47.1)	4249 (45.0)	0.79
Available toilet facility			
Sanitary/semi-sanitary	12,289 (93.6)	8979 (95.1)	Ref.
Non-sanitary	840 (6.4)	461 (4.9)	<0.001
Common pathogens isolated			
*Cryptosporidium*	843 (6.4)	1123 (11.9)	<0.001
*Giardia*	3470 (26.4)	1786 (18.9)	<0.001
*Entamoeba histolytica*	299 (2.3)	279 (2.9)	<0.001
ETEC	975 (7.4)	1067 (11.3)	<0.001
*Campylobacter*	1561 (11.9)	1171 (12.4)	0.63
EAEC	2655 (20.2)	1846 (19.6)	0.33
*Shigella*	231 (1.8)	1110 (11.8)	<0.001
Common viral pathogens isolated			
Rotavirus	509 (3.9)	1747 (18.5)	<0.001
Norovirus	962 (7.3)	741 (7.8)	0.149
Adenovirus	98 (0.7)	235 (2.5)	<0.001
Astrovirus	261 (2.0 %)	238 (2.5 %)	0.008
Sapovirus	456 (3.5 %)	325 (3.4 %)	0.933

ETEC: enterotoxigenic *E. coli*; EAEC: enteroaggregative *E. coli*; HAZ/LAZ: height/length-for-age, WAZ: weight-for-age, and WHZ: weight-for-height z-scores; Ref.: reference.

a Mean ± SD (standard deviation).

**Table 2:** WASH situation and practices among the symptomatic MSD children having stool positive for viral pathogens in South Asia and sub-Saharan Africa.

c	Rotavirus			Norovirus			Adenovirus			Astrovirus			Sapovirus		
					
n (%)	Negative	Positive	P-value	Negative	Positive	P-value	Negative	Positive	P-value	Negative	Positive	P-value	Negative	Positive	P-value
															
	7693 (%)	1747 (%)		8697 (%)	743 (%)		9205 (%)	235 (%)		9201 (%)	239 (%)		9115 (%)	325 (%)	

Household toilet facility															
Sanitary/semi sanitary	7298 (94.9)	1681 (96.2)	0.02	8272 (95.1)	707 (95.2)	1	8759 (95.2)	220 (93.6)	0.35	8749 (95.1)	230 (96.2)	0.51	8665 (95.1)	314 (96.6)	0.25
Non-sanitary	395 (5.1)	66 (3.8)		425 (4.9)	36 (4.8)		446 (4.8)	15 (6.4)		452 (4.9)	9 (3.8)		450 (4.9)	11 (3.4)	
Hand washing															
Without soap	5851 (76.1)	1280 (73.3)	0.01	6596 (75.8)	535 (72.0)	0.02	6967 (75.7)	164 (69.8)	0.05	6959 (75.6)	172 (72.0)	0.22	6893 (75.6)	238 (73.2)	0.36
With soap and water	1842 (23.9)	466 (26.7)		2100 (24.1)	208 (28.0)		2237 (24.3)	71 (30.2)		2241 (24.4)	67 (28.0)		2221 (24.4)	87 (26.8)	
Handwash before nurse or preparing baby food															
No	4783 (62.2)	974 (55.8)	<0.001	5352 (61.5)	405 (54.5)	<0.001	5629 (61.2)	128 (54.5)	0.05	5638 (61.3)	119 (49.8)	<0.001	5573 (61.1)	184 (56.6)	0.11
Yes	2910 (37.8)	773 (44.2)		3345 (38.5)	338 (45.5)		3576 (38.8)	107 (45.5)		3563 (38.7)	120 (50.2)		3542 (38.9)	141 (43.4)	
Handwash after cleaning a child who defecated															
No	4215 (54.8)	976 (55.9)	0.43	4836 (55.6)	355 (47.8)	<0.001	5075 (55.1)	116 (49.4)	0.09	5078 (55.2)	113 (47.3)	0.02	5042 (55.3)	149 (45.8)	<0.001
Yes	3478 (45.2)	771 (44.1)		3861 (44.4)	388 (52.2)		4130 (44.9)	119 (50.6)		4123 (44.8)	126 (52.7)		4073 (44.7)	176 (54.2)	
Wash hands before cooking															
No	2998 (39.0)	683 (39.1)	0.95	3396 (39.0)	285 (38.4)	0.74	3593 (39.0)	88 (37.4)	0.67	3604 (39.2)	77 (32.2)	0.04	3568 (39.1)	113 (34.8)	0.13
Yes	4695 (61.0)	1064 (60.9)		5301 (61.0)	458 (61.6)		5612 (61.0)	147 (62.6)		5597 (60.8)	162 (67.8)		5547 (60.9)	212 (65.2)	
Wash hands after handling animal															
No	7036 (91.5)	1623 (92.9)	0.05	7991 (91.9)	668 (89.9)	0.07	8442 (91.7)	217 (92.3)	0.82	8432 (91.6)	227 (95.0)	0.08	8357 (91.7)	302 (92.9)	0.49
Yes	657 (8.5)	124 (7.1)		706 (8.1)	75 (10.1)		763 (8.3)	18 (7.7)		769 (8.4)	12 (5.0)		758 (8.3)	23 (7.1)	
The main source of drinking water															
Tube well water	1397 (18.2)	278 (15.9)	0.03	1541 (17.7)	134 (18.0)	0.87	1629 (17.7)	46 (19.6)	0.51	1645 (17.9)	30 (12.6)	0.04	1641 (18.0)	34 (10.5)	<0.001
Non-tube well water	6296 (81.8)	1469 (84.1)		7156 (82.3)	609 (82.0)		7576 (82.3)	189 (80.4)		7556 (82.1)	209 (87.4)		7474 (82.0)	291 (89.5)	
Drinking water container mouth															
Narrow	831 (10.8)	239 (13.7)	<0.001	983 (11.3)	87 (11.7)	0.47	1037 (11.3)	33 (14.0)	0.12	1041 (11.3)	29 (12.1)	0.49	1038 (11.4)	32 (9.8)	1
Wide	4971 (64.6)	1049 (60.0)		5572 (64.1)	448 (60.3)		5885 (63.9)	135 (57.4)		5881 (63.9)	139 (58.2)		5839 (64.1)	181 (55.7)	
Water container covered															
No	997 (13.0)	211 (12.1)	0.54	1129 (13.0)	79 (10.6)	0.14	1177 (12.8)	31 (13.2)	1	1187 (12.9)	21 (8.8)	0.15	1186 (13.0)	22 (6.8)	0.004
Yes	5432 (70.6)	1213 (69.4)		6125 (70.4)	520 (70.0)		6477 (70.4)	168 (71.5)		6481 (70.4)	164 (68.6)		6414 (70.4)	231 (71.1)	
Water trips daily (mean, SD)	4.24 (3.52)	4.07 (3.2)	0.14	4.24 (3.5)	3.78 (2.8)	0.002	4.21 (3.5)	3.91 (2.99)	0.24	4.22 (3.5)	3.49 (2.8)	0.01	4.22 (3.5)	3.73 (2.9)	0.04
Time to retrieve drinking water															
Less than 15 min	4166 (54.2)	906 (51.9)	0.001	4742 (54.5)	330 (44.4)	<0.001	4932 (53.6)	140 (59.6)	0.01	4969 (54.0)	103 (43.1)	0.05	4933 (54.1)	139 (42.8)	0.01
15 min and more	1669 (21.7)	449 (25.7)		1908 (21.9)	210 (28.3)		2083 (22.6)	35 (14.9)		2058 (22.4)	60 (25.1)		2035 (22.3)	83 (25.5)	
Fetch drinking water every day															
No	1506 (19.6)	331 (18.9)	0.31	1691 (19.4)	146 (19.7)	0.44	1800 (19.6)	37 (15.7)	0.21	1791 (19.5)	46 (19.2)	0.48	1774 (19.5)	63 (19.4)	0.37
Yes	4331 (56.3)	1024 (58.6)		4961 (57.0)	394 (53.0)		5217 (56.7)	138 (58.7)		5238 (56.9)	117 (49.0)		5196 (57.0)	159 (48.9)	
Water availability															
All the time	5198 (67.6)	1045 (59.8)	<0.001	5779 (66.4)	464 (62.4)	0.03	6112 (66.4)	131 (55.7)	<0.001	6105 (66.4)	138 (57.7)	0.01	6054 (66.4)	189 (58.2)	0.002
Not all the time	2494 (32.4)	702 (40.2)		2917 (33.5)	279 (37.6)		3092 (33.6)	104 (44.3)		3095 (33.6)	101 (42.3)		3060 (33.6)	136 (41.8)	
Given stored water for drinking															
No	1965 (25.5)	429 (24.6)	0.42	2209 (25.4)	185 (24.9)	0.80	2333 (25.3)	61 (26.0)	0.89	2337 (25.4)	57 (23.8)	0.64	2326 (25.5)	68 (20.9)	0.07
Yes	5728 (74.5)	1317 (75.4)		6487 (74.6)	558 (75.1)		6871 (74.6)	174 (74.0)		6863 (74.6)	182 (76.2)		6788 (74.5)	257 (79.1)	
Drinking water retrieval method															
Pour (spigot or spout)															
No	4025 (52.3)	893 (51.1)	0.34	4552 (52.3)	366 (49.3)	0.17	4807 (52.2)	111 (47.2)	0.14	4793 (52.1)	125 (52.3)	0.98	4746 (52.1)	172 (52.9)	0.23
Yes	2969 (38.6)	696 (39.8)		3362 (38.7)	303 (40.8)		3563 (38.7)	102 (43.4)		3573 (38.8)	92 (38.5)		3555 (39.0)	110 (33.8)	
Scoop with cup															
No	2445 (31.8)	601 (34.4)	0.03	2792 (32.1)	254 (34.2)	0.18	2973 (32.3)	73 (31.1)	0.77	2974 (32.3)	72 (30.1)	0.52	2972 (32.6)	74 (22.8)	0.001
Yes	4552 (59.2)	989 (56.6)		5125 (58.9)	416 (56.0)		5401 (58.7)	140 (59.6)		5396 (58.6)	145 (60.7)		5333 (58.5)	208 (64.0)	
Scoop with ladle															
No	6911 (89.8)	1564 (89.5)	0.22	7815 (89.9)	660 (88.8)	0.77	8267 (89.8)	208 (88.5)	0.28	8264 (89.8)	211 (88.3)	0.30	8200 (90.0)	275 (84.6)	0.13
Yes	85 (1.1)	26 (1.5)		101 (1.2)	10 (1.3)		106 (1.2)	5 (2.1)		106 (1.2)	5 (2.1)		104 (1.1)	7 (2.2)	

**Table 3:** WASH situation and practices among the asymptomatic children having stool positive for viral pathogens in South Asia and sub-Saharan Africa.

WASH indicators	Rotavirus			Norovirus			Adenovirus			Astrovirus			Sapovirus		
					
n (%)	Negative	Positive	P-value	Negative	Positive	P-value	Negative	Positive	P-value	Negative	Positive	P-value	Negative	Positive	P-value
													
	12,620 (%)	509 (%)		12,167 (%)	962 (%)		13,031 (%)	98 (%)		12,868 (%)	261 (%)		12,673 (%)	456 (%)	

Household toilet facility															
Sanitary/semi sanitary	11,803 (93.5)	486 (95.5)	0.09	11,384 (93.6)	905 (94.1)	0.58	12,202 (93.6)	87 (88.8)	0.08	12,041 (93.6)	248 (95.0)	0.41	11,854 (93.5)	435 (95.4)	0.14
Non-sanitary	817 (6.5)	23 (4.5)		783 (6.4)	57 (5.9)		829 (6.4)	11 (11.2)		827 (6.4)	13 (5.0)		819 (6.5)	21 (4.6)	
Hand washing material															
Without soap	9392 (74.4)	370 (72.7)	0.45	9044 (74.3)	718 (74.6)	0.87	9686 (74.3)	76 (77.6)	0.54	9583 (74.5)	179 (68.6)	0.04	9427 (74.4)	335 (73.5)	0.69
With soap and water	3227 (25.6)	138 (27.1)		3121 (25.7)	244 (25.4)		3343 (25.7)	22 (22.4)		3283 (25.5)	82 (31.4)		3244 (25.6)	121 (26.5)	
Handwash before nurse or preparing baby food															
No	7733 (61.3)	263 (51.7)	<0.001	7386 (60.7)	610 (63.4)	0.11	7943 (61.0)	53 (54.1)	0.20	7837 (60.9)	159 (60.9)	1	7697 (60.7)	299 (65.6)	0.04
Yes	4887 (38.7)	246 (48.3)		4781 (39.3)	352 (36.6)		5088 (39.0)	45 (45.9)		5031 (39.1)	102 (39.1)		4976 (39.3)	157 (34.4)	
After cleaning a child who defecated															
No	6682 (52.9)	258 (50.7)	0.34	6407 (52.7)	533 (55.4)	0.11	6891 (52.9)	49 (50.0)	0.64	6805 (52.9)	135 (51.7)	0.76	6690 (52.8)	250 (54.8)	0.42
Yes	5938 (47.1)	251 (49.3)		5760 (47.3)	429 (44.6)		6140 (47.1)	49 (50.0)		6063 (47.1)	126 (48.3)		5983 (47.2)	206 (45.2)	
Wash hands before cooking															
No	3866 (30.6)	126 (24.8)	0.01	3695 (30.4)	297 (30.9)	0.77	3958 (30.4)	34 (34.7)	0.41	3899 (30.3)	93 (35.6)	0.07	3872 (30.6)	120 (26.3)	0.06
Yes	8754 (69.4)	383 (75.2)		8472 (69.6)	665 (69.1)		9073 (69.6)	64 (65.3)		8969 (69.7)	168 (64.4)		8801 (69.4)	336 (73.7)	
Wash hands after handling animal															
No	11,174 (88.5)	437 (85.9)	0.07	10,747 (88.3)	864 (89.8)	0.18	11,528 (88.5)	83 (84.7)	0.32	11,386 (88.5)	225 (86.2)	0.30	11,202 (88.4)	409 (89.7)	0.44
Yes	1446 (11.5)	72 (14.1)		1420 (11.7)	98 (10.2)		1503 (11.5)	15 (15.3)		1482 (11.5)	36 (13.8)		1471 (11.6)	47 (10.3)	
The main source of drinking water															
Tube well	2890 (22.9)	104 (20.4)	0.21	2799 (23.0)	195 (20.3)	0.06	2965 (22.8)	29 (29.6)	0.14	2943 (22.9)	51 (19.5)	0.23	2915 (23.0)	79 (17.3)	0.01
Non-tube well	9730 (77.1)	405 (79.6)		9368 (77.0)	767 (79.7)		10,066 (77.2)	69 (70.4)		9925 (77.1)	210 (80.5)		9758 (77.0)	377 (82.7)	
Drinking water container mouth															
Narrow	1495 (11.8)	107 (21.0)	<0.001	1477 (12.1)	125 (13.0)	0.22	1583 (12.1)	19 (19.4)	0.06	1576 (12.2)	26 (10.0)	0.50	1558 (12.3)	44 (9.6)	0.20
Wide	8344 (66.1)	306 (60.1)		8052 (66.2)	598 (62.2)		8589 (65.9)	61 (62.2)		8485 (65.9)	165 (63.2)		8355 (65.9)	295 (64.7)	
Water container covered															
No	1963 (15.6)	92 (18.1)	0.09	1918 (15.8)	137 (14.2)	0.44	2035 (15.6)	20 (20.4)	0.28	2028 (15.8)	27 (10.3)	0.05	2004 (15.8)	51 (11.2)	0.04
Yes	8905 (70.6)	337 (66.2)		8578 (70.5)	664 (69.0)		9176 (70.4)	66 (67.3)		9059 (70.4)	183 (70.1)		8928 (70.4)	314 (68.9)	
Time to retrieve drinking water															
Less than 15 min	6552 (51.9)	238 (46.8)	<0.001	6383 (52.5)	407 (42.3)	<0.001	6743 (51.7)	47 (48.0)	0.10	6678 (51.9)	112 (42.9)	0.003	6587 (52.0)	203 (44.5)	0.12
15 min and more	2901 (23.0)	180 (35.4)		2829 (23.3)	252 (26.2)		3049 (23.4)	32 (32.7)		3002 (23.3)	79 (30.3)		2970 (23.4)	111 (24.3)	
Fetch drinking water every day															
No	1956 (15.5)	85 (16.7)	0.93	1872 (15.4)	169 (17.6)	0.001	2018 (15.5)	23 (23.5)	0.09	2004 (15.6)	37 (14.2)	0.72	1965 (15.5)	76 (16.7)	0.13
Yes	7493 (59.4)	332 (65.2)		7336 (60.3)	489 (50.8)		7769 (59.6)	56 (57.1)		7671 (59.6)	154 (59.0)		7588 (59.9)	237 (52.0)	
Water trips per day (mean, SD)	4.48 (3.3)	5.55 (3.7)	<0.001	4.53 (3.3)	4.51 (3.1)	0.87	4.53 (3.3)	4.82 (2.5)	0.40	4.52 (3.3)	4.75 (3.3)	0.41	4.54 (3.3)	4.29 (2.9)	0.21
Water availability															
All the time	8724 (69.1)	341 (67.0)	0.33	8435 (69.3)	630 (65.5)	0.02	8995 (69.0)	70 (71.4)	0.69	8899 (69.2)	166 (63.6)	0.06	8749 (69.0)	316 (69.3)	0.95
Not all the time	3896 (30.9)	168 (33.0)		3732 (30.7)	332 (34.5)		4036 (31.0)	28 (28.6)		3969 (30.8)	95 (36.4)		3924 (31.0)	140 (30.7)	
Given stored water for drinking															
No	4124 (32.7)	162 (31.8)	0.74	3970 (32.6)	316 (32.8)	0.93	4250 (32.6)	36 (36.7)	0.45	4204 (32.7)	82 (31.4)	0.75	4152 (32.8)	134 (29.4)	0.14
Yes	8489 (67.3)	346 (68.0)		8189 (67.3)	646 (67.2)		8773 (67.3)	62 (63.3)		8657 (67.3)	178 (68.2)		8513 (67.2)	322 (70.6)	
Drinking water retrieval method															
Pour (spigot or spout)															
No	6368 (50.5)	262 (51.5)	0.31	6154 (50.6)	476 (49.5)	0.77	6587 (50.5)	43 (43.9)	0.27	6498 (50.5)	132 (50.6)	0.60	6381 (50.4)	249 (54.6)	0.01
Yes	5537 (43.9)	206 (40.5)		5322 (43.7)	421 (43.8)		5695 (43.7)	48 (49.0)		5637 (43.8)	106 (40.6)		5579 (44.0)	164 (36.0)	
Scoop with cup															
No	4727 (37.5)	205 (40.3)	0.09	4550 (37.4)	382 (39.7)	0.09	4887 (37.5)	45 (45.9)	0.08	4830 (37.5)	102 (39.1)	0.37	4780 (37.7)	152 (33.3)	0.22
Yes	7184 (56.9)	264 (51.9)		6932 (57.0)	516 (53.6)		7402 (56.8)	46 (46.9)		7312 (56.8)	136 (52.1)		7187 (56.7)	261 (57.2)	
Scoop with ladle															
No	11,790 (93.4)	462 (90.8)	0.74	11,371 (93.5)	881 (91.6)	0.01	12,164 (93.3)	88 (89.8)	0.10	12,017 (93.4)	235 (90.0)	0.97	11,844 (93.5)	408 (89.5)	0.90
Yes	121 (1.0)	6 (1.2)		110 (0.9)	17 (1.8)		124 (1.0)	3 (3.1)		124 (1.0)	3 (1.1)		122 (1.0)	5 (1.1)	

## Data Availability

A publicly available GEMS dataset was analyzed in this study. This data can be obtained here: ClinEpiDB (https://clinepidb.org/ce/app/workspace/analyses/DS_841a9f5259/new/variables/PCO_0000024/ENVO_00000009). Following the thorough review and approval process by the ClinEpiDB team, we have obtained official data access from ClinEpiDB, the responsible entity for managing the GEMS data repository.

## HIGHLIGHTS

- Characterize the role of WASH exposures and practices as risk factors for specific enteric viral infections in children under 5.
- Norovirus and Astrovirus were detected more commonly in sub-Saharan Africa while Rotavirus was less prevalent than in South Asia
- Found no significant association between poor sanitation conditions and different enteric viral pathogens examined.
- Though significant associations were found with WASH indicators, no overall pattern between WASH and enteric viral pathogens was observed.
