# Supplementary material for: Impact of water, sanitation, and hygiene indicators on enteric viral pathogens among under-5 children in low resource settings
Source: Sci Total Environ. Author manuscript; Available in PMC 2025 Apr 15. (PMC11999324; doi:10.1016/j.scitotenv.2025.178401)
Supplement: Supplementary file 2 [file NIHMS2065949-supplement-Supplementary_file_2.docx]

**Supplementary Table 2.** Baseline characteristics of the asymptomatic children having stool positive for viral pathogens in South Asia and sub-Saharan Africa

| **Characteristics** | **Rotavirus** | | | **Norovirus** | | | **Adenovirus** | | | **Astrovirus** | | | **Sapovirus** | | |
| --- | --- | --- | --- | --- | --- | --- | --- | --- | --- | --- | --- | --- | --- | --- | --- |
| **n (%)** | **Negative** | **Positive** | **P-value** | **Negative** | **Positive** | **P-value** | **Negative** | **Positive** | **P-value** | **Negative** | **Positive** | **P-value** | **Negative** | **Positive** | **P-value** |
|  | **12620(%)** | **509(%)** |  | **12167(%)** | **962(%)** |  | **13031(%)** | **98(%)** |  | **12868(%)** | **261(%)** |  | **12673(%)** | **456(%)** |  |
| **Age group** |  |  |  |  |  |  |  |  |  |  |  |  |  |  |  |
| 0-11 m | 4634 (36.7) | 244 (47.9) | <0.001 | 4511 (37.1) | 367 (38.1) | 0.101 | 4837 (37.1) | 41 (41.8) | 0.007 | 4775 (37.1) | 103 (39.5) | 0.738 | 4700 (37.1) | 178 (39.0) | 0.006 |
| 12-23 m | 4208 (33.3) | 173 (34.0) |  | 4041 (33.2) | 340 (35.3) |  | 4339 (33.3) | 42 (42.9) |  | 4297 (33.4) | 84 (32.2) |  | 4208 (33.2) | 173 (37.9) |  |
| 24-59 m | 3778 (29.9) | 92 (18.1) |  | 3615 (29.7) | 255 (26.5) |  | 3855 (29.6) | 15 (15.3) |  | 3796 (29.5) | 74 (28.4) |  | 3765 (29.7) | 105 (23.0) |  |
| **Gender** |  |  |  |  |  |  |  |  |  |  |  |  |  |  |  |
| Boy | 7182 (56.9) | 296 (58.2) | 0.61 | 6922 (56.9) | 556 (57.8) | 0.609 | 7424 (57.0) | 54 (55.1) | 0.787 | 7305 (56.8) | 173 (66.3) | 0.003 | 7202 (56.8) | 276 (60.5) | 0.129 |
| Girl | 5438 (43.1) | 213 (41.8) |  | 5245 (43.1) | 406 (42.2) |  | 5607 (43.0) | 44 (44.9) |  | 5563 (43.2) | 88 (33.7) |  | 5471 (43.2) | 180 (39.5) |  |
| **Breastfeeding status** | |  |  |  |  |  |  |  |  |  |  |  |  |  |  |
| No | 3971 (31.5) | 119 (23.4) | <0.001 | 3789 (31.1) | 301 (31.3) | 0.953 | 4066 (31.2) | 24 (24.5) | 0.187 | 4001 (31.1) | 89 (34.1) | 0.332 | 3964 (31.3) | 126 (27.6) | 0.109 |
| Yes | 8649 (68.5) | 390 (76.6) |  | 8378 (68.9) | 661 (68.7) |  | 8965 (68.8) | 74 (75.5) |  | 8867 (68.9) | 172 (65.9) |  | 8709 (68.7) | 330 (72.4) |  |
| **Wealth quintile** |  |  |  |  |  |  |  |  |  |  |  |  |  |  |  |
| Poorest | 2413 (19.1) | 97 (19.1) | 0.508 | 2335 (19.2) | 175 (18.2) | 0.257 | 2491 (19.1) | 19 (19.4) | 0.785 | 2460 (19.1) | 50 (19.2) | 0.897 | 2437 (19.2) | 73 (16.0) | 0.397 |
| Lower-middle | 2492 (19.7) | 98 (19.3) |  | 2396 (19.7) | 194 (20.2) |  | 2575 (19.8) | 15 (15.3) |  | 2544 (19.8) | 46 (17.6) |  | 2494 (19.7) | 96 (21.1) |  |
| Middle | 2712 (21.5) | 122 (24.0) |  | 2601 (21.4) | 233 (24.2) |  | 2811 (21.6) | 23 (23.5) |  | 2773 (21.5) | 61 (23.4) |  | 2727 (21.5) | 107 (23.5) |  |
| Upper-middle | 2437 (19.3) | 85 (16.7) |  | 2343 (19.3) | 179 (18.6) |  | 2500 (19.2) | 22 (22.4) |  | 2473 (19.2) | 49 (18.8) |  | 2430 (19.2) | 92 (20.2) |  |
| Richest | 2565 (20.3) | 107 (21.0) |  | 2491 (20.5) | 181 (18.8) |  | 2653 (20.4) | 19 (19.4) |  | 2617 (20.3) | 55 (21.1) |  | 2584 (20.4) | 88 (19.3) |  |
| **Baseline Anthropometry** | |  |  |  |  |  |  |  |  |  |  |  |  |  |  |
| **HAZ** | -1.34 (1.31) | -1.39 (1.34) | 0.379 | -1.33 (1.31) | -1.51 (1.35) | <0.001 | -1.34 (1.31) | -1.30 (1.15) | 0.683 | -1.34 (1.31) | -1.44 (1.37) | 0.25 | -1.34 (1.31) | -1.48 (1.38) | 0.033 |
| **WAZ** | -1.08 (1.32) | -0.90 (1.33) | 0.003 | -1.06 (1.31) | -1.33 (1.33) | <0.001 | -1.08 (1.32) | -1.13 (1.47) | 0.733 | -1.08 (1.32) | -1.14 (1.32) | 0.475 | -1.07 (1.32) | -1.30 (1.34) | <0.001 |
| **WHZ** | -0.479 (1.43) | -0.17 (1.45) | <0.001 | -0.45 (1.43) | -0.68 (1.41) | <0.001 | -0.47 (1.43) | -0.58 (1.71) | 0.514 | -0.47 (1.43) | -0.48 (1.44) | 0.868 | -0.46 (1.43) | -0.70 (1.36) | <0.001 |

^¥^ mean and SD (standard deviation); HAZ: height for age z score; WAZ: weight for age z score; WHZ: weight for height z score; breastfeed: exclusive and partial breastfeeding; MSD: moderate-to-severe diarrhoea.
