# Supplementary material for: Impact of water, sanitation, and hygiene indicators on enteric viral pathogens among under-5 children in low resource settings
Source: Sci Total Environ. Author manuscript; Available in PMC 2025 Apr 15. (PMC11999324; doi:10.1016/j.scitotenv.2025.178401)
Supplement: Supplementary file 3 [file NIHMS2065949-supplement-Supplementary_file_3.docx]

**Supplementary Table 3.** Impact of WASH indicators on symptomatic MSD children having stool positive for viral pathogens (Rotavirus, norovirus, adenovirus, astrovirus, and sapovirus) in South Asia and sub-Saharan Africa.

|  | **WASH indicators** | **Rotavirus** | **Norovirus** | **Adenovirus** | **Astrovirus** | **Sapovirus** |
| --- | --- | --- | --- | --- | --- | --- |
|  |  | **aOR (95% CI)** | **aOR (95% CI)** | **aOR (95% CI)** | **aOR (95% CI)** | **aOR (95% CI)** |
| **The main source of drinking water** | | |  |  |  |  |
|  | Tube well water | Reference | | | | |
|  | Non-tube well water | 1.23(0.96,1.6) | 0.82(0.58,1.18) | **0.47(0.27,0.86) *** | 0.87(0.48,1.64) | 1.22(0.7,2.22) |
| **Time to retrieve drinking water** | |  |  |  |  |  |
|  | Less than 15 minutes | Reference |  |  |  |  |
|  | 15 minutes and more | 1.09(0.94,1.25) | **1.33(1.08,1.64) *** | 1.04(0.72,1.5) | **1.43(1.01,2.02) *** | 0.97(0.71,1.33) |
| **Drinking water retrieval method** | |  |  |  |  |  |
| **Pour (spigot or spout)** | |  |  |  |  |  |
|  | No | Reference |  |  |  |  |
|  | Yes | 0.98(0.79,1.2) | 0.74(0.55,1.01) | 1.47(0.9,2.4) | 0.62(0.37,1.02) | 0.87(0.55,1.36) |
| **Scoop with cup** | |  |  |  |  |  |
|  | No | Reference |  |  |  |  |
|  | Yes | **0.77(0.62,0.96) *** | **0.75(0.55,1.04) *** | **2.3(1.32,4.11) *** | 0.75(0.45,1.27) | 1.39(0.85,2.31) |
| **Water availability** | |  |  |  |  |  |
|  | All the time | Reference |  |  |  |  |
|  | Not all the time **^¥^** | **1.2(1.01,1.42) *** | 1.19(0.92,1.52) | 1.39(0.88,2.17) | 1.37(0.91,2.06) | 1.13(0.77,1.64) |
| **The child was given stored water for drinking** | | |  |  |  |  |
|  | No | Reference |  |  |  |  |
|  | Yes | 1.13(0.92,1.39) | 1.04(0.77,1.43) | 1.28(0.75,2.28) | 1.07(0.65,1.84) | 1.19(0.75,1.95) |
| **Toilet facility** | |  |  |  |  |  |
|  | Sanitary/ semi sanitary | Reference |  |  |  |  |
|  | Non-sanitary | 0.92(0.67,1.24) | 1.07(0.69,1.6) | 0.82(0.32,1.73) | 0.84(0.32,1.78) | 1.62(0.88,2.76) |
| **Handwashing material** | |  |  |  |  |  |
|  | With soap and water | Reference |  |  |  |  |
|  | Without soap | **0.85(0.73,0.99) *** | 1.17(0.94,1.46) | 0.95(0.64,1.38) | 1.18(0.81,1.7) | 0.89(0.63,1.25) |
| **Handwashing practice** | | |  |  |  |  |
| **Before nurse or prepare baby food** | | |  |  |  |  |
|  | No | Reference |  |  |  |  |
|  | Yes | **1.21(****1.06,1.4) *** | **1.24(****1.01,1.52) *** | 1.23(0.86,1.75) | 1.24(0.87,1.76) | 1.23(0.9,1.67) |
| **After cleaning a child who defecated** | | |  |  |  |  |
|  | No | Reference |  |  |  |  |
|  | Yes | 0.91(0.8,1.03) | 1.12(0.93,1.36) | 1.09(0.79,1.52) | 1.21(0.87,1.68) | 1.17(0.88,1.56) |
| **Before cooking** | |  |  |  |  |  |
|  | No | Reference |  |  |  |  |
|  | Yes | 0.97(0.85,1.11) | 0.96(0.8,1.17) | 1.27(0.9,1.8) | 1.01(0.72,1.42) | 0.95(0.71,1.28) |
| **After handling animal** | | |  |  |  |  |
|  | No | Reference |  |  |  |  |
|  | Yes | 0.81(0.62,1.04) | 1.32(0.95,1.81) | 1.24(0.68,2.13) | 0.46(0.18,1.003) | 1.34(0.77,2.24) |
| **Continent** | |  |  |  |  |  |
|  | South Asia | Reference |  |  |  |  |
|  | sub-Saharan Africa | 1.18 (0.99, 1.41) | 1.09 (0.84, 1.41) | 1.32 (0.84, 2.08) | 1.23 (0.80, 1.88) | 1.22 (0.82, 1.81) |

Adjusted for age, gender, and continent; Separate multiple logistic regression models were performed to see the association of enteric viral pathogens with WASH, where dependent variables were enteric viral pathogens (Rotavirus, norovirus, adenovirus, astrovirus, and sapovirus).

*P value <0.05; Abbreviation: aOR (adjusted odds ratio), CI: confidence interval.

**^¥^** Not all the time: Several hours every day to less frequently than a few times per week
