# Supplementary material for: Impact of water, sanitation, and hygiene indicators on enteric viral pathogens among under-5 children in low resource settings
Source: Sci Total Environ. Author manuscript; Available in PMC 2025 Apr 15. (PMC11999324; doi:10.1016/j.scitotenv.2025.178401)
Supplement: Supplementary file 4 [file NIHMS2065949-supplement-Supplementary_file_4.docx]

**Supplementary Table 4.** Impact of WASH indicators on asymptomatic children having stool positive for viral pathogens (Rotavirus, norovirus, adenovirus, astrovirus, and sapovirus) in South Asia and sub-Saharan Africa.

|  | **WASH indicators** | **Rotavirus** | **Norovirus** | **Adenovirus** | **Astrovirus** | **Sapovirus** |
| --- | --- | --- | --- | --- | --- | --- |
|  |  | **aOR (95% CI)** | **aOR (95% CI)** | **aOR (95% CI)** | **aOR (95% CI)** | **aOR (95% CI)** |
| **The main source of drinking water** | | |  |  |  |  |
|  | Tube well water | Reference |  |  |  |  |
|  | Non-tube well water | 0.77(0.55,1.09) | **1.38(1.01,1.89) *** | 0.88(0.38,2.17) | 1.34(0.77,2.38) | 1.43(0.91,2.26) |
| **Time to retrieve drinking water** | |  |  |  |  |  |
|  | Less than 15 minutes | Reference |  |  |  |  |
|  | 15 minutes and more | **1.51(1.2,1.89) *** | **1.47(1.21,1.78) *** | 1.37(0.78,2.39) | 1.37(0.97,1.93) | 1.11(0.84,1.45) |
| **Drinking water retrieval method** | |  |  |  |  |  |
| **Pour (spigot or spout)** | |  |  |  |  |  |
|  | No | Reference |  |  |  |  |
|  | Yes | 0.75(0.52,1.07) | 0.86(0.65,1.13) | 1.9 (0.9,4.4) | **0.51(0.32,0.83) *** | 0.68(0.45,1.03) |
| **Scoop with cup** | |  |  |  |  |  |
|  | No | Reference |  |  |  |  |
|  | Yes | **0.51(0.35,0.73) *** | 0.78(0.59,1.04) | 1.35(0.59,3.11) | **0.52(0.32,0.86) *** | 0.92(0.6,1.44) |
| **Water availability** | |  |  |  |  |  |
|  | All the time | Reference |  |  |  |  |
|  | Not all the time **^¥^** | **1.57(1.21,2.02) *** | 1.01(0.8,1.26) | 1.45(0.75,2.73) | 1.11(0.74,1.64) | 0.88(0.64,1.22) |
| **The child was given stored water for drinking** | | |  |  |  |  |
|  | No | Reference |  |  |  |  |
|  | Yes | 1.06(0.8,1.43) | 0.84(0.66,1.08) | 0.95(0.48,2.01) | 1.02(0.66,1.61) | 0.87(0.62,1.23) |
| **Toilet facility** | |  |  |  |  |  |
|  | Sanitary/ semi sanitary | Reference |  |  |  |  |
|  | Non-sanitary | 0.91(0.56,1.39) | 1.07(0.76,1.46) | 0.76(0.23,1.89) | 0.73(0.34,1.38) | 0.85(0.49,1.4) |
| **Handwashing material** | |  |  |  |  |  |
|  | With soap and water | Reference |  |  |  |  |
|  | Without soap | 1.22(0.96,1.53) | 0.83(0.68,1.01) | 1.23(0.71,2.07) | 1.25(0.89,1.73) | 0.82(0.61,1.08) |
| **Hand washing practice** | |  |  |  |  |  |
| **Before the nurse/ prepares baby food** | | |  |  |  |  |
|  | No | Reference |  |  |  |  |
|  | Yes | **1.48(****1.18,1.85) *** | 1.04(0.86,1.24) | 0.79(0.46,1.35) | 0.95(0.68,1.32) | 0.88(0.67,1.15) |
| **After cleaning a child who defecated** | | |  |  |  |  |
|  | No | Reference |  |  |  |  |
|  | Yes | 0.89(0.71,1.11) | 0.95(0.8,1.13) | 0.72(0.43,1.18) | 1.26(0.92,1.73) | 1.07(0.84,1.38) |
| **Before cooking** | |  |  |  |  |  |
|  | No | Reference |  |  |  |  |
|  | Yes | **1.5(****1.17,1.94) *** | 0.87(0.73,1.04) | 1.11(0.66,1.91) | **0.64(****0.47,0.88) *** | 1.14(0.87,1.5) |
| **After handling animal** | | |  |  |  |  |
|  | No | Reference |  |  |  |  |
|  | Yes | 1.17(0.86,1.58) | 1.01(0.78,1.3) | 1.51(0.75,2.9) | 1.32(0.83,2.04) | 1.12(0.77,1.61) |
| **Continent** | |  |  |  |  |  |
|  | South Asia | Reference |  |  |  |  |
|  | Sub-Saharan Africa | **0.46 (0.35, 0.60) *** | **1.46(1.17,1.82) *** | 0.81(0.43,1.53) | **1.47(1.01,2.17) *** | 1.33(0.96,1.84) |

Adjusted for age, gender, and continent; Separate multiple logistic regression models were performed to see the association of enteric viral pathogens with WASH, where dependent variables were enteric viral pathogens (Rotavirus, norovirus, adenovirus, astrovirus, and sapovirus).

*P value <0.05; Abbreviation: aOR (adjusted odds ratio), CI: confidence interval.

**^¥^** Not all the time: Several hours every day to less frequently than a few times per week
