# Supplementary material for: Impact of water, sanitation, and hygiene indicators on enteric viral pathogens among under-5 children in low resource settings
Source: Sci Total Environ. Author manuscript; Available in PMC 2025 Apr 15. (PMC11999324; doi:10.1016/j.scitotenv.2025.178401)
Supplement: Supplementary file 1 [file NIHMS2065949-supplement-Supplementary_file_1.docx]

**Supplementary Table 1.** Baseline characteristics of the symptomatic MSD children having stool positive for viral pathogens in South Asia and sub-Saharan Africa

| **Characteristics** | **Rotavirus** | | | **Norovirus** | | | **Adenovirus** | | | **Astrovirus** | | | **Sapovirus** | | |
| --- | --- | --- | --- | --- | --- | --- | --- | --- | --- | --- | --- | --- | --- | --- | --- |
| **n (%)** | **Negative** | **Positive** | **P-value** | **Negative** | **Positive** | **P-value** | **Negative** | **Positive** | **P-value** | **Negative** | **Positive** | **P-value** | **Negative** | **Positive** | **P-value** |
|  | **7693 (%)** | **1747 (%)** |  | **8697(%)** | **743(%)** |  | **9205 (%)** | **235(%)** |  | **9201(%)** | **239(%)** |  | **9115(%)** | **325(%)** |  |
| **Age group** |  |  |  |  |  |  |  |  |  |  |  |  |  |  |  |
| 0-11 m | 3015 (39.2) | 1015 (58.1) | <0.001 | 3686 (42.4) | 344 (46.3) | 0.038 | 3908 (42.5) | 122 (51.9) | <0.001 | 3906 (42.5) | 124 (51.9) | 0.002 | 3891 (42.7) | 139 (42.8) | 0.007 |
| 12-23 m | 2633 (34.2) | 572 (32.7) |  | 2954 (34.0) | 251 (33.8) |  | 3114 (33.8) | 91 (38.7) |  | 3126 (34.0) | 79 (33.1) |  | 3074 (33.7) | 131 (40.3) |  |
| 24-59 m | 2045 (26.6) | 160 (9.2) |  | 2057 (23.7) | 148 (19.9) |  | 2183 (23.7) | 22 (9.4) |  | 2169 (23.6) | 36 (15.1) |  | 2150 (23.6) | 55 (16.9) |  |
| **Gender** |  |  |  |  |  |  |  |  |  |  |  |  |  |  |  |
| Boy | 4386 (57.0) | 959 (54.9) | 0.113 | 4940 (56.8) | 405 (54.5) | 0.241 | 5217 (56.7) | 128 (54.5) | 0.543 | 5209 (56.6) | 136 (56.9) | 0.981 | 5167 (56.7) | 178 (54.8) | 0.530 |
| Girl | 3307 (43.0) | 788 (45.1) |  | 3757 (43.2) | 338 (45.5) |  | 3988 (43.3) | 107 (45.5) |  | 3992 (43.4) | 103 (43.1) |  | 3948 (43.3) | 147 (45.2) |  |
| **Breastfeeding status** |  |  |  |  |  |  |  |  |  |  |  |  |  |  |  |
| No | 2455 (31.9) | 243 (13.9) | <0.001 | 2485 (28.6) | 213 (28.7) | 0.992 | 2658 (28.9) | 40 (17.0) | <0.001 | 2632 (28.6) | 66 (27.6) | 0.792 | 2599 (28.5) | 99 (30.5) | 0.484 |
| Yes | 5237 (68.1) | 1504 (86.1) |  | 6211 (71.4) | 530 (71.3) |  | 6546 (71.1) | 195 (83.0) |  | 6568 (71.4) | 173 (72.4) |  | 6515 (71.5) | 226 (69.5) |  |
| **Wealth quintile** |  |  |  |  |  |  |  |  |  |  |  |  |  |  |  |
| Poorest | 1641 (21.3) | 386 (22.1) | 0.908 | 1851 (21.3) | 176 (23.7) | 0.196 | 1975 (21.5) | 52 (22.1) | 0.211 | 1973 (21.4) | 54 (22.6) | 0.234 | 1958 (21.5) | 69 (21.2) | 0.661 |
| Lower-middle | 1486 (19.3) | 327 (18.7) |  | 1675 (19.3) | 138 (18.6) |  | 1780 (19.3) | 33 (14.0) |  | 1771 (19.2) | 42 (17.6) |  | 1744 (19.1) | 69 (21.2) |  |
| Middle | 1633 (21.2) | 360 (20.6) |  | 1840 (21.2) | 153 (20.6) |  | 1937 (21.0) | 56 (23.8) |  | 1932 (21.0) | 61 (25.5) |  | 1924 (21.1) | 69 (21.2) |  |
| Upper-middle | 1449 (18.8) | 331 (18.9) |  | 1628 (18.7) | 152 (20.5) |  | 1728 (18.8) | 52 (22.1) |  | 1733 (18.8) | 47 (19.7) |  | 1716 (18.8) | 64 (19.7) |  |
| Richest | 1479 (19.2) | 342 (19.6) |  | 1697 (19.5) | 124 (16.7) |  | 1779 (19.3) | 42 (17.9) |  | 1786 (19.4) | 35 (14.6) |  | 1768 (19.4) | 53 (16.3) |  |
| **Baseline anthropometry** | |  |  |  |  |  |  |  |  |  |  |  |  |  |  |
| **HAZ** | -1.37 (1.35) | -1.18 (1.44) | <0.001 | -1.33 (1.37) | -1.40 (1.29) | 0.153 | -1.34 (1.36) | -1.13 (1.39) | 0.023 | -1.33 (1.37) | -1.43 (1.32) | 0.263 | -1.34 (1.36) | -1.33 (1.40) | 0.933 |
| **WAZ** | -1.53 (1.39) | -1.43 (1.40) | 0.006 | -1.50 (1.39) | -1.62 (1.35) | 0.024 | -1.52 (1.39) | -1.25 (1.42) | 0.005 | -1.51 (1.39) | -1.63 (1.36) | 0.157 | -1.51 (1.39) | -1.45 (1.41) | 0.402 |
| **WHZ** | -1.07 (1.49) | -1.03 (1.50) | 0.328 | -1.06 (1.49) | -1.16 (1.44) | 0.058 | -1.07 (1.49) | -0.87 (1.50) | 0.049 | -1.06 (1.49) | -1.14 (1.56) | 0.438 | -1.07 (1.49) | -0.973 (1.47) | 0.260 |

^¥^ mean and SD (standard deviation); HAZ: height for age z score; WAZ: weight for age z score; WHZ: weight for height z score; breastfeed: exclusive and partial breastfeeding; MSD: moderate-to-severe diarrhoea.
